# Evaluating distinct KRAS subtypes as potential biomarkers for immune checkpoint inhibitor efficacy in lung adenocarcinoma

**DOI:** 10.3389/fimmu.2023.1297588

**Published:** 2023-10-24

**Authors:** Qi Wang, Zhuoran Tang, Chunyu Li, Xuefei Li, Chunxia Su

**Affiliations:** ^1^ Department of Medical Oncology, Shanghai Pulmonary Hospital, School of Medicine, Tongji University, Shanghai, China; ^2^ School of Medicine, Tongji University, Shanghai, China; ^3^ Department of Integrated Traditional Chinese and Western Medicine, International Medical School, Tianjin Medical University, Tianjin, China; ^4^ Department of Lung Cancer and Immunology, Shanghai Pulmonary Hospital, School of Medicine, Tongji University, Shanghai, China

**Keywords:** KRAS, subtype, immune checkpoint inhibitors (ICIs), lung adenocarcinoma (LUAD), tumor immune microenvironment

## Abstract

**Background:**

Despite the acknowledged predictive value of KRAS in immune checkpoint inhibitor (ICI) responses, the heterogeneous behavior of its mutations in this sphere remains largely unexplored. As of now, no studies have definitively categorized KRAS subtype variations as independent prognostic indicators for ICI responses in lung cancer patients.

**Methods:**

We analyzed a cohort of 103 patients, all harboring different KRAS mutation subtypes, and complemented this data with information from TCGA and GEO databases. Our research focused on delineating the relationships between KRAS mutation subtypes and factors like immunotherapy markers and immune cell composition, in addition to examining survival rates, drug sensitivity, and PD-L1 responses corresponding to distinct KRAS subtypes.

**Results:**

We found that the G12V and G12D subtypes demonstrated elevated expressions of immunotherapy markers, implying a potentially enhanced benefit from immunotherapy. Significant variations were identified in the distribution of naive B cells, activated CD4+ memory T cells, and regulatory T cells (Tregs) across different KRAS mutant subtypes. A notable difference was observed in the Tumor Mutation Burden (TMB) levels across the four KRAS subtypes, with the G12D subtype displaying the lowest TMB level. Furthermore, G12C subtype showcased the worst prognosis in terms of progression-free intervals (PFI), in stark contrast to the more favorable outcomes associated with the G12A subtype.

**Conclusion:**

Our study reveals that KRAS mutations exhibit considerable variability in predicting outcomes for LUAD patients undergoing ICI treatment. Thus, the evaluation of KRAS as a biomarker for ICIs necessitates recognizing the potential diversity inherent in KRAS mutations.

## 1 Introduction

Immune checkpoint inhibitors (ICIs) have emerged as a potent frontier in cancer treatment, demonstrating promising potential in combatting various malignancies (1, 2). Particularly in the context of lung adenocarcinoma, ICIs herald a new era of therapeutic possibilities (3). Despite the enthusiasm surrounding the clinical impact of ICIs, it is imperative to acknowledge that a significant portion of patients remain unresponsive to this form of treatment, underscoring the pressing need for efficacious biomarkers.

Current research indicates that PD-L1, TMB, and IFN-γ stand as credible biomarkers in predicting ICI responses (4, 5). However, the reliance on expensive panels for the accurate detection of TMB and immune signatures presents a significant obstacle. Consequently, the scientific community is pivoting towards more accessible methodologies, like next-generation sequencing (NGS), to facilitate the identification of genomic alterations that could potentially dictate patient responsiveness to ICIs (2). This approach aims to streamline the process of pinpointing individuals who are likely to benefit from ICI treatments, fostering a more targeted and cost-effective therapeutic strategy.

Numerous genetic variations have been identified as having a correlation with the efficacy of immune checkpoint inhibitors (ICIs), encompassing mutations found in genes such as EGFR, ALK, KRAS, TP53, STK11, JAK2, and ATM (4, 6). Within this array, the KRAS gene, a member of the ras gene family, stands as one of the most frequently mutated oncogenes in non-small cell lung cancer (NSCLC). Traditionally, KRAS has been dubbed an “undruggable target,” evading the grasp of effective targeted therapies, thereby necessitating a focus on driver gene negative NSCLC for the long-term treatment of patients harboring KRAS mutations (7).

Recent primary and clinical research endeavors have embarked on a detailed exploration of the immune microenvironment characteristics and the clinical outcomes of immunotherapy in patients with KRAS mutations (8–10). Analyses of clinical samples from this patient demographic revealed heightened levels of Tumor Mutation Burden (TMB), PD-L1 expression, and tumor-infiltrating lymphocytes (11).

While existing literature hints at a significant association between KRAS mutations and a heightened immunogenicity within the tumor and inflammatory microenvironment, suggesting a potential favorable response to ICI therapy, the precise impact on prognosis remains inadequately elucidated. Furthermore, the predictive value of these mutations concerning patient survival outcomes is yet to be firmly established (10, 12).

Existing research indicates that while KRAS mutations as a whole might not be robust indicators of patient outcomes, a deeper analysis into the distinct subtypes of this mutation — as well as their coexistence with mutations in other genes — could potentially offer a richer insight into patient prognosis and inform subsequent immunotherapy strategies (13). It is plausible that NSCLC featuring KRAS mutations constitutes a heterogeneous spectrum of diseases, each harboring unique molecular subtypes. This underlines the necessity for a comprehensive appraisal of these subtypes in the context of clinical treatments. At this juncture, KRAS mutation subtypes are not recognized as standalone predictors for responses to ICIs. Given the functional diversity inherent to KRAS mutations, we propose the hypothesis that their predictive power concerning ICI efficacy may also be distinctly varied. Consequently, a structured and detailed categorization of the diverse mutant KRAS variants is imperative to leverage their full potential as predictive biomarkers in clinical settings.

## 2 Method

### 2.1 mRNA expression profiling and analysis from public datasets

RNA-seq data was available for 563 LUAD patients within the TCGA database. We utilized resources like the cBioPortal and the TCPA from the Cancer Genome Atlas to obtain protein array data pertinent to cancer studies. For the purpose of correlation analysis, gene expression data was extracted employing the appropriate R package. Furthermore, the Java GSEA Desktop Application can be accessed at http://software.broadinstitute.org/gsea/index.jsp to facilitate the use of GSEA in linking genetic markers to KRAS mutations. To illustrate the functional pathways effectively, a point plot was generated using the ClusterProfiler tool within the R programming environment.

### 2.2 Data sources

RNA-seq data, somatic mutation information, and immunotherapy data specific to lung adenocarcinoma were retrieved from the TCGA subpopulation and the MSK cohort pertaining to lung cancer. From the extracted somatic mutation data, seven distinct KRAS mutation subtypes were identified. This included limited sample subsets such as five samples exclusively identifying with G12S, five with G13C, and three with G13D. Given the insufficient sample sizes of these three subtypes, they were deemed unsuitable for subsequent statistical analysis. Consequently, the study focused on the four primary mutation subtypes: G12A, G12C, G12D, and G12V, which presented a more substantial data pool for comprehensive investigation.

### 2.3 Drug sensitivity analysis

The R package was used to predict drug IC50 values for TCGA RNA-seq samples. It mainly indicates IC50 values of samples using two drug databases, the cancer therapeutics response portal (CTRP) and genomics of drug sensitivity in cancer (GDSC). The IC50 values of 148 lung cancer drugs were analyzed using the database of GDSC version V2.0.

### 2.4 NGS-based assay

Following the protocol, DNA was isolated from FFPE tumor samples utilizing the QIAamp DNA FFPE Tissue Kit and the Tissue Extraction Kit from Qiagen. To guarantee a tumor content exceeding 70%, expert pathologists meticulously examined the FFPE tissue specimens. Meanwhile, DNA from peripheral blood was obtained using Qiagen’s DNEasy Blood and the QIAamp DNA Blood Mini Kit, facilitating the purification of genomic DNA from 2 ml of peripheral blood samples.

The integrity and quality of the extracted genomic DNA, both from tumor tissues and peripheral blood, were assessed through agarose gel electrophoresis. The Agilent 2100 Bioanalyzer, equipped with a DNA 1000 Kit, facilitated the evaluation of the size dispersion of the circulating DNA fragments. To determine the purity and concentration levels of the DNA samples, instruments such as the NanoDrop2000 spectrophotometer and the Qubit 2.0 fluorometer were employed in conjunction with a dsDNA HS Assay Kit supplied by Yeasen China.

To maintain rigorous quality control throughout the testing phase, each assay incorporated a minimum of one positive control, one negative control, and one blank control. Concurrently, routine samples were processed, establishing and adhering to stringent quality control standards.

### 2.5 Patients and clinical information

We conducted a retrospective study, in which a total of 103 individuals diagnosed with stage III-IV and KRAS mutation NSCLC at Shanghai Pulmonary Hospital from January 2018 to November 2022, their tissue and peripheral blood samples were routinely subjected to next-generation sequencing (NGS) to check for specific genomic alterations before mono-immunotherapy. Only individuals with measurable diseases, and subsequent image studies available for response assessment, were selected for this research. This study was a retrospective single-center study to explore the response and recurrence after PD-1/PD-L1 monoclonal antibody treatment. In addition to overall response rate (ORR), progression-free survival (PFS), and overall survival (OS). Oncology Group Performance Status (ECOG PS) 0 or 1. Clinical data were extracted from the electronic patient record system. Information, including patients’ age, gender, ethnicity, pathology, and tumor stage, was collected (**Table 1**). The hospital’s Ethics Committee granted its approval for this study.

**Table 1:** Baseline patient characteristics.

Characteristic	N (%)
Gender	
Male	47 (45.6%)
Female	56 (54.4%)
Age (years)	
≥50	83 (80.6%)
<50	20 (19.4%)
TNM stage	
I-IIIA	11 (10.7%)
IIIB-IV	92 (89.3%)
Smoking Status	
No	49 (47.6%)
Yes	54 (52.4%)
KRAS Subtype	
G12A	12 (11.7%)
G12V	22 (21.4%)
G12C	46 (44.7%)
G12D	23 (22.2%)
ECOG	
1	98 (95.1%)
2	5 (4.9%)

### 2.6 Statistical analysis

Data analysis was conducted utilizing GraphPad Prism 9 (GraphPad Software, Inc.) and R version 4.0 (R Foundation for Statistical Computing). Statistical significance was determined using Fisher’s exact test. To compare progression-free survival rates, we utilized the log-rank test available in GraphPad Prism 9, facilitating the creation of Kaplan-Meier survival curves. In this study, a p-value less than 0.05 was deemed statistically significant, with all tests being two-sided. A p-value less than 0.1 was considered to be marginally significant.

### 2.7 Single cell RNA-seq data analysis

In this study, we sourced public single-cell RNA sequencing data of colorectal cancer from the NCBI Gene Expression Omnibus (GEO) database, under the accession code GSE132465. Following quality control procedures, we proceeded with standard normalization and unsupervised clustering utilizing Seurat V4. This involved the application of functions such as ‘NormalizeData’, ‘FindVariableFeatures’, and ‘ScaleData’. Dimensionality reduction analyses were facilitated through Principal Component Analysis (PCA) and Uniform Manifold Approximation and Projection (UMAP). We executed cell clustering utilizing the ‘FindClusters’ function, which adopts the shared nearest neighbor modularity optimization-based clustering algorithm at a resolution of 0.2. We then conducted differential gene expression (DEG) analysis between varying cell clusters, utilizing the Wilcoxon rank-sum test as implemented in the ‘FindAllMarkers’ function within Seurat. Criteria for determining DEGs were genes exhibited in over 25% of cells, a log2 fold change exceeding 0.25 compared to the background, and a false discovery rate below 0.05. Upon identifying the top DEGs for each cluster, we annotated the cell types using the deCS package. Additionally, KRAS mutation data was extracted from its corresponding whole-exome sequencing (WES) data present in the original study. To scrutinize each cell type further, we analyzed the fold change of immune checkpoint genes using the Wilcoxon test.

## 3 Results

### 3.1 Association of KRAS mutation subtypes with immunotherapy and immune checkpoint expression metrics

RNA-seq data, somatic mutation details, and immunotherapy information specific to lung adenocarcinoma were extracted from the TCGA subpopulation and the MSK lung cancer cohort. KRAS mutation subtypes were analyzed for associations with common immunotherapy indicators, including PD-L1, PD-L2, PD-1, CYT, and GEP. Previous studies suggest that GEP and CYT of T cell inflammation are emerging as predictive biomarkers for PD-1 blockade therapy. Our study found that the expression differences of immunotherapy indicators were not particularly significant among the overall four subtypes. However, from the results of our analysis, higher expression of immunotherapy indicators was observed in the G12V, and G12D subtypes, which illustrates the potential for the greater clinical benefit of immunotherapy targeting patients with KRAS mutations in both subtypes (**Supplementary Figures 1A–E**).

**Figure 1:**
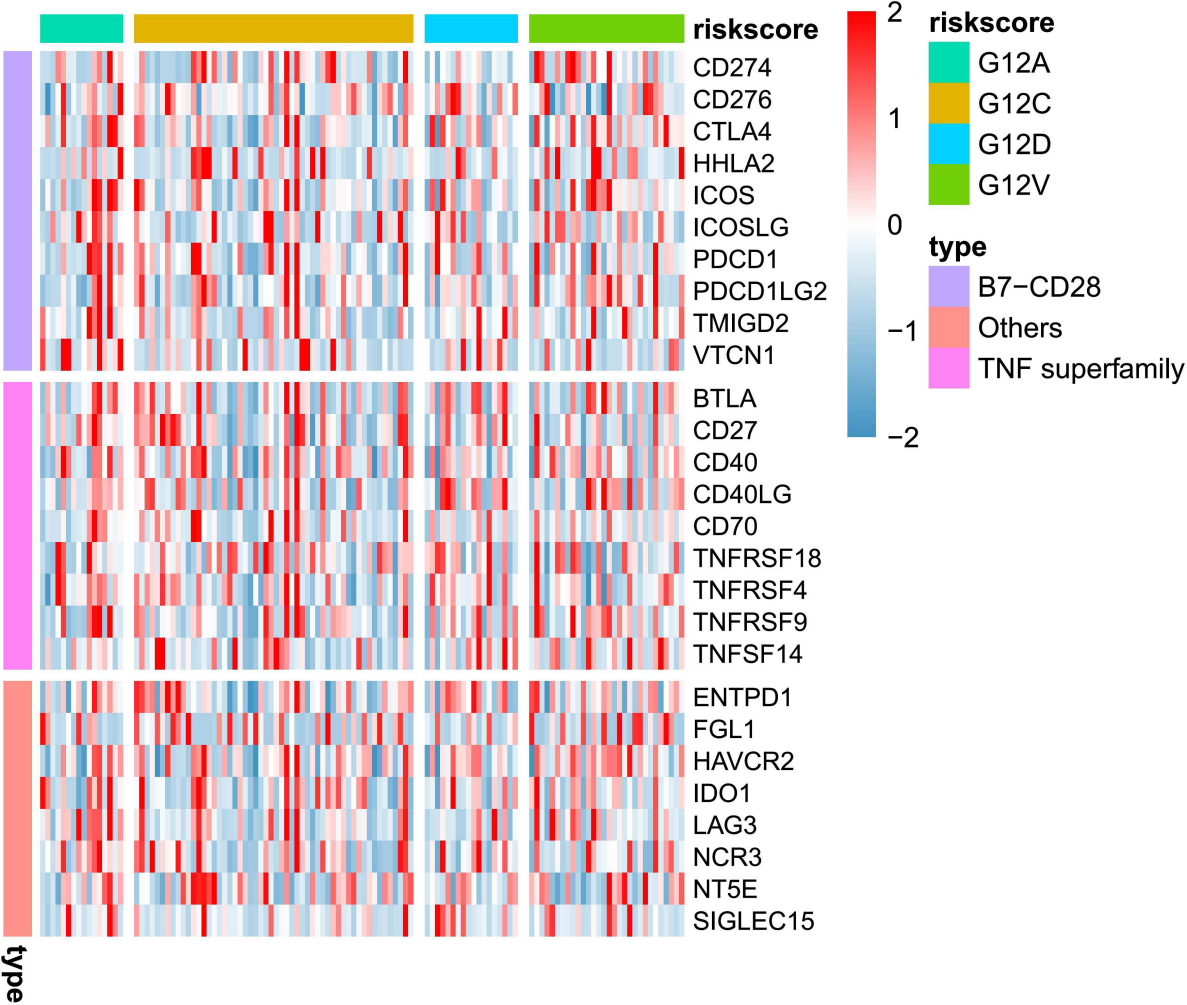
Heatmap illustrates the distribution of immune checkpoint gene expressions across various KRAS mutation subtypes.

Expression heat maps of immune checkpoint genes were plotted for different subtypes of KRAS mutations (**Figure 1**). The results showed that G12C overall immune checkpoint expression was lower and that each KRAS isoform was differentially expressed with high immune checkpoint expression. The above results, illustrate that different therapeutic targets should be selected for different KRAS subtypes.

### 3.2 Single-cell analysis of immune checkpoint gene expression in KRAS mutant vs. wildtype patients

**Figure 2:**
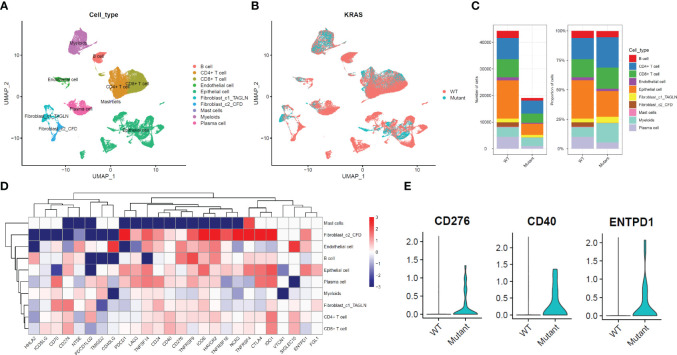
Expression Changes of Immune Checkpoint Genes at the Single-Cell Level in Colorectal Cancer with KRAS mutation. **(A)** UMAP plot displaying single-cell RNA sequencing data, categorized by cell type. **(B)** UMAP plot categorizing single-cell RNA sequencing data based on KRAS mutation status. **(C)** Comparison of total cell count and cell type composition between WT (Wild-Type) and KRAS mutant patient groups. **(D)** Differential gene expression analysis of immune checkpoint genes at the cell type level. **(E)** Violin plot illustrating significantly higher expression levels of CD276, CD40, and ENTPD1 in the fibroblast_c2_CFD group. For the comparison of those three genes, a student’s t-test was conducted with a P-value < 0.0001.

While bulk RNA-seq has indicated that various KRAS mutants may influence the response to immune therapy, a more detailed analysis is necessary to substantiate this, ideally at the single-cell level. In **Figure 2A**, we present an overview representation of scRNA-seq based on publicly available data of colorectal cancer. A total of 10 cell types were detected, including two distinct fibroblast clusters. The UMAP analysis confirmed that KRAS mutation demonstrate certain level of impact for most cell type. We observed from the UMAP plot that KRAS mutations have a certain degree of impact on specific cell types, especially epithelial (malignant tumor) and fibroblast populations (**Figure 2B**). Next, we assessed the proportions of major cell types across between WT and KRAS mutant group. As showed in **Figure 2C**, we observed the increase of CD4+ T cell and myeloid cells in KRAS mutant group. In contrast, we notice the plasma cells, as well as fibroblast cluster 2 (fibroblast c2) where this particular subpopulation appeared to decrease, even diminish in KRAS mutant group.

To further explore the impact of KRAS mutation for different cell types, we conducted a comprehensive DEG analysis on immune checkpoint genes (The selection of these genes is same in **Figure 1**). As shown in **Figure 2D**, we noticed most checkpoint genes were up-regulated in fibroblast c2 and epithelial (malignant tumor). For example, CD276, CD40, ENTPD1 demonstrated significant up regulated in KRAS mutant group (**Figure 2E**).

### 3.3 Differences in the distribution of 22 immune cell types across different subtypes of KRAS

To further explore the differences between the different mutational subtypes of KRAS, we utilized the R package cluster profiler (v3.16.1) to perform GO, KEGG, and GSEA functional enrichment analyses on the KRAS mutation subtype data. Used the R package Mitch to analyze differences in the hallmark functions of KRAS subtypes. We found that the G12A and G12V subtypes were more enriched in interferon related response, and that G12V was enriched in inflammatory response, TNF-α signaling via NF-kB (**Supplementary Figures 2A–G**).

**Figure 3:**
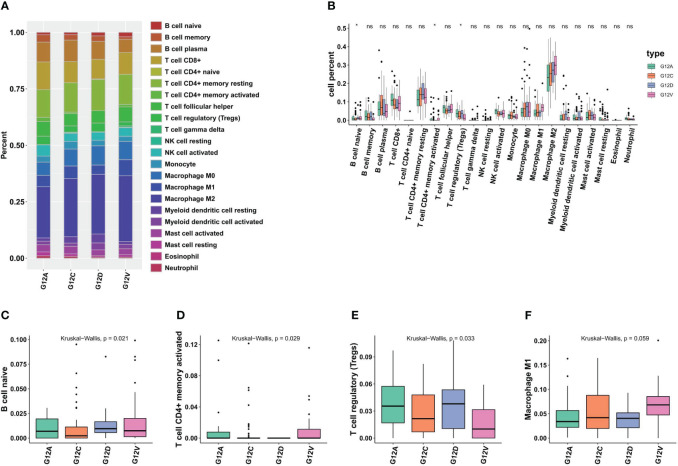
Different KRAS mutation subtypes are associated with different immune cell composition and tumor microenvironment. **(A)** The bar plots illustrate the varying cell composition percentages among different KRAS mutation subtypes. **(B)** Statistical analysis reveals the extent of significant differences in the Tumor Microenvironment (TME) among four KRAS mutation subtypes. **(C–E)** Boxplots illustrate significant variations in the proportions of naive B cells, CD4+ memory cells, Tregs, and M1 macrophages across the four KRAS mutations. *, *p*<0.05; ns, not significant.

TILs (tumor-infiltrating lymphocytes) exhibit pro- and anti-tumor properties. Tregs, tumor-associated macrophages (TAMs; M2), and other cells linked to immunotherapy side effects and pre-tumor function. We wanted to find out how immune cells were distributed among the various KRAS mutant populations in LUAD tumors. Based on extensive RNA expression data, a deconvolution approach for cell type enrichment analysis was used to calculate the immune cell level (**Figures 3A, B**). B cells are Naive, T cells have CD4 ^+^ memory activation, Tregs and other immune cells differ significantly between the KRAS mutant subtypes (**Figures 3C–F**). The findings additionally suggest that tumor tissues harboring distinct KRAS mutations exhibit variations in immune cell composition.

### 3.4 Differences in TMB, DNA damage repair defects between different mutational subtypes of KRAS

**Figure 4:**
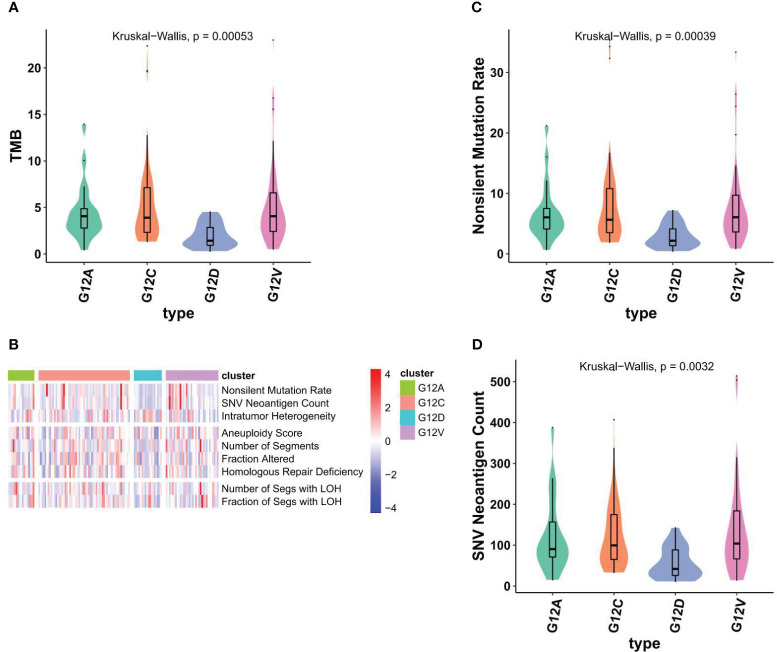
Different KRAS mutation subtypes are associated with TMB and nonsilent mutation rate. **(A)** Violin plots showed TMBs are associated with KRAS subtypes. **(B)** The comprehensive heatmap displays the variations in DNA alterations across individual KRAS subtypes. **(C, D)** violin plots showed nonsilent mutation rate **(C)** and SNV neoantigen counts across individual KRAS subtypes **(D)**.

The cellular stress response caused by DNA damage is essential for ensuring the stability of genetic material, inhibiting the generation of gene mutations, and maintaining the life span of cells. As in other malignancies, the development of NSCLC is a multifactorial, multistage, and multistep complex process. The primary mechanism result from various factors leading to proto-oncogene activation or tumor suppressor gene inactivation, hypofunction or deletion of DNA damage repair genes, and the joint participation of some signaling pathways. DNA damage repair defects included DNA mutations (Nonsilent mutation rate, SNV), copy number variations (Aneurysm Score, number of segments, fraction altered, homologous repair deficiency), loss of heterozygosity (number of SEGS with LOH, number of SEGS with LOH). We aimed to investigate if distinct KRAS mutation subtypes correspond to differing mutation burdens. Notably, patients harboring the G12C mutation demonstrate a significantly elevated overall mutation rate (**Figures 4A, B**), while The G12D subtype has the lowest TMB level. Those mutations are all defined as nonsilent mutations through our analysis (**Figures 4C**). We next also determined which DDR pathway was associated with KRAS mutation status and whether DDR deletion led to significant increases in TMB and neoantigen levels in different KRAS states. The analysis results showed that there were significant differences in SNV neoantigen counts among the four KRAS mutant subtypes (**Figure 4D**).

### 3.5 Prognostic differences among different KRAS mutation subtypes in the TCGA lung cancer database

**Figure 5:**
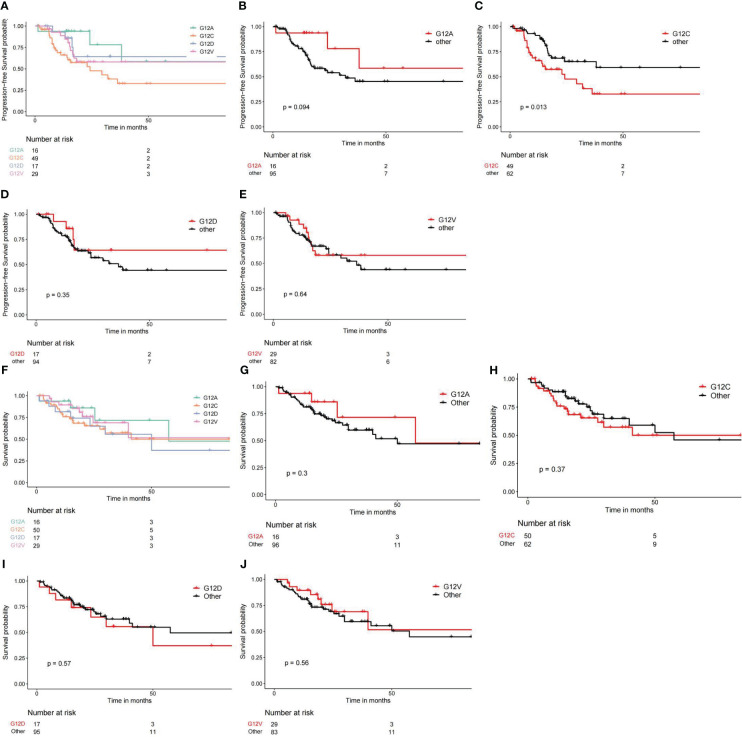
Different KRAS mutation subtypes lead to different survival outcomes. **(A–E)** Comparing PFI or PFS from different KRAS subtypes. **(F–J)** Comparing overall survival outcomes across different KRAS subtypes.

We conducted an analysis of the prognostic disparities among various subtypes of KRAS mutations utilizing the “survival” package in R. Subsequently, Kaplan-Meier survival curves were constructed with the assistance of the “survminer” package in R. The analysis of Progression-Free Intervals (PFI) revealed that individuals with the G12C mutation faced the poorest prognosis, while those with the G12A mutation exhibited the most favorable outcomes (**Figures 5A–E**). Moreover, the statistical analysis underscored a significant difference in PFI results when G12C was compared with other subtypes. In terms of overall survival (OS), the trend persisted with G12C representing the worst prognosis and G12A denoting the best. However, it is noteworthy that the differences in OS prognosis among the various subtypes were not statistically significant, as depicted in **Figures 5F–J**.

### 3.6 LUAD cases with the KRAS G12A mutation demonstrate a more favorable response to anti-PD-1/L1 therapy compared to those possessing the KRAS G12C mutation

Utilizing the R package, we estimated the IC50 values for TCGA RNAseq samples, leveraging two prominent drug databases: the Cancer Therapeutics Response Portal (CTRP) and the Genomics of Drug Sensitivity in Cancer (GDSC). For our analysis, we opted for the GDSC database’s Version 2 to scrutinize the IC50 values associated with 148 lung cancer pharmaceuticals. Notably, AZ6102 stands out as a potent inhibitor of TNKS1/2, exhibiting a hundredfold selectivity over other enzymes in the PARP family. Statistical analysis revealed significant differences between the IC50 values obtained for different KRAS mutant subtypes targeting AZ6102 (**Supplementary Figures 3A–C**).

Our previous analyses have highlighted the distinct impacts of variances and similarities in factors such as PD-L1 expression, TMB, and TME within the KRAS subgroup on the efficacy of ICI therapy. Consequently, we sought to determine whether LUAD cases exhibiting different KRAS mutations respond differently to anti-PD-1/L1 therapy. In this study, we included 103 well-balanced patients who were free of EGFR and ALK gene variants and had received first-line immunotherapy. The patient cohort, which received ICI treatment between June 2018 and November 2022, was characterized by well-confirmed KRAS mutant subtypes as determined through NGS sequencing. Initially, the breakdown of patients with measurable lung tumors was as follows: 12 with KRAS G12A, 22 with KRAS G12V, 46 with KRAS G12C, and 23 with KRAS G12D.

**Figure 6:**
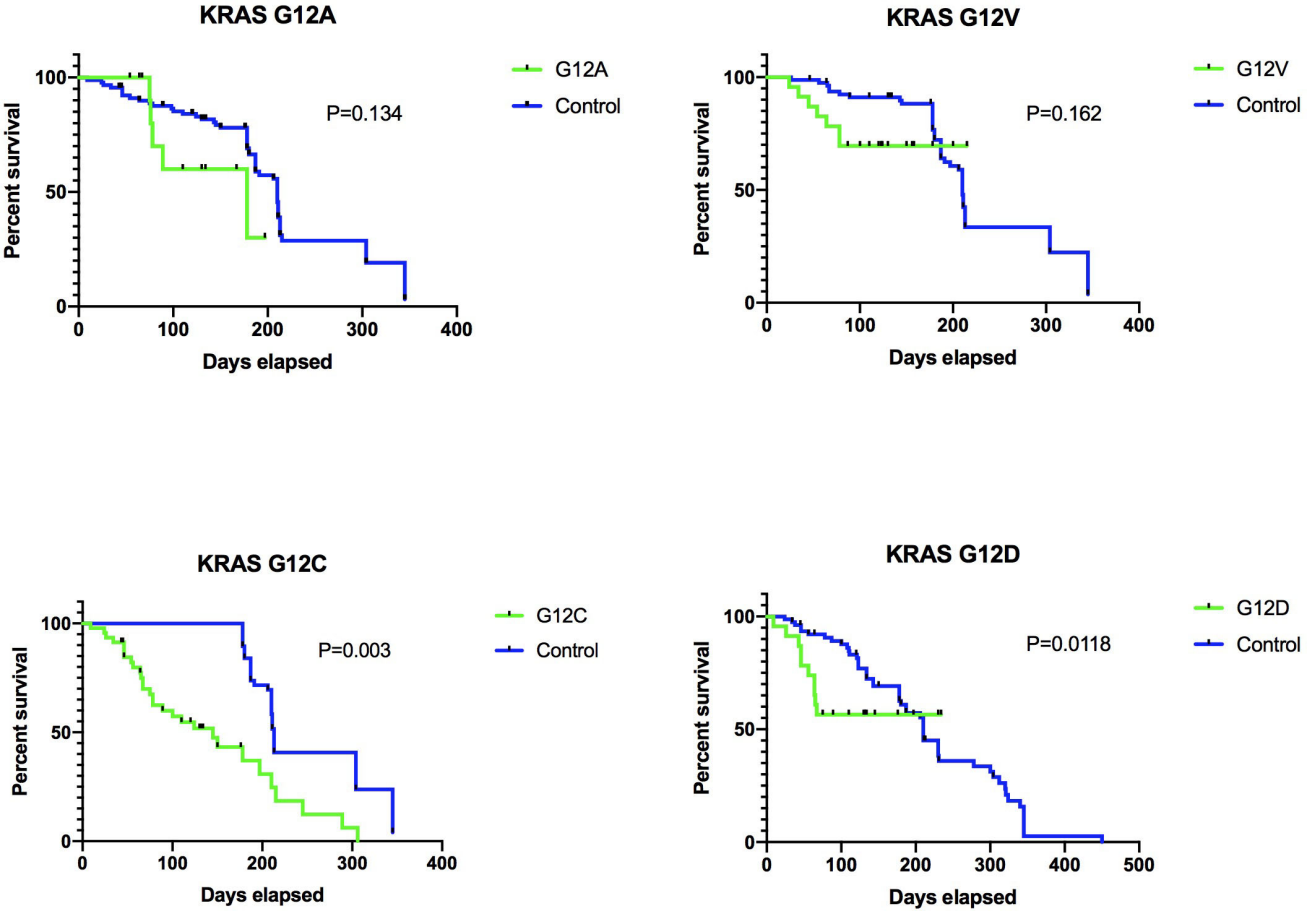
Various KRAS mutation subtypes result in distinct PD-L1 responses and affect different cohorts. Our data is derived from an in-house cohort of 103 individuals. The breakdown of these mutation subtypes is as follows: G12A, n=12 (11.7%); G12V, n=22 (21.4%); G12C, n=46 (44.7%); G12D, n=23 (22.2%). The Kaplan-Meier analysis was employed to evaluate the survival outcomes between the two groups.

Upon evaluation for treatment efficacy, data revealed a notable divergence in progression-free survival (PFS) rates between groups. Specifically, the KRAS G12A group exhibited a significantly extended PFS—6.5 months—compared to the 4.7 months observed in the KRAS G12C group, a difference substantiated by a log-rank p-value of 0.003 (**Figure 6**). In conclusion, our clinical data analysis indicates that the KRAS subtype serves as a distinct marker in forecasting the responsiveness to ICI therapy. Specifically, LUADs harboring the KRAS G12C mutation did not exhibit any enhanced clinical benefits from ICI treatment.

## 4 Discussion

Tumor immunity is a new pillar in cancer treatment today, and it plays a revolutionary role in the treatment of cancer. The ICI is composed of PD-1 and PD-L1, and its function is to unleash the tumor-suppressive immune system (14–16). Although there have been some breakthroughs, ICI treatment is not entirely without side effects, and it is unacceptable to every patient. Therefore, it is necessary to find biomarkers that can effectively identify the therapeutic effects of ICIs. The clinically applied biomarkers mainly include PD-L1 immunohistochemistry and high instability-high, MSI-H or error repair (dMMR) (17–19). Pembrolizumab(anti-PD-1) was approved by the U.S. Food and Drug Administration (FDA) in 2017 to treat advanced MSI-H/dMMR solid tumors that have progressed after prior treatment, regardless of tumor type. The FDA has never before approved a molecular biomarker regardless of the type of malignancy. Even though PD-L1 and MSI-H/dMMR are both regarded as indicators of ICI response (17, 20). They still lack stripes, however, and they have limited sensitivity and specificity. As a result, there is a need to keep looking for biomarkers that can more accurately predict how well an ICI will respond to treatment. One such indicator is tumor mutation burden (TMB). High TMB is linked to ICI responsiveness in several tumor types (21). For instance, in patients treated with nivolumab and ipilimumab, high TMB was associated with significantly improved progression-free survival in non-small cell lung cancer (NSCLC) regardless of PD-L1 expression (22).

There were significant differences in the expression of TMB and PD-L1 in tumor genes. A systematic meta-analysis showed that patients with KRAS gene variants benefited from anti-PD-1/PD-L1 immunotherapy (23). The immunogenicity of cancer is usually caused by gene mutation, and the greater the amount of mutation of TMB, the stronger its immunogenicity. However, dMMR can also contribute to tumor immunogenicity, and both genes may be favorable populations for immunotherapy. Mutations in the KRAS gene are related to the microenvironment of inflammatory tumors and the immunogenicity of tumors, making patients respond better to PD-1 inhibitors (12). Different types of KRAS variants were associated with treatment outcomes, while specific gene variants were destined not to benefit from immunotherapy. The expression levels of PD-L1 and TMB genes are still unknown in different KRAS gene mutation subtype.

Our results showed that TMBs of four common KRAS variants significantly differed in TMB expression. TMB content was lowest in the G12D subtype. Several recent studies have shown that TMB can act as a surrogate to replace the entire new antigen load, and disruption of DNA damage repair pathways causes an increase in TMB. In non-small cell lung cancer, TMB is the most effective biomarker for predicting immune-checkpoint blockade (ICB) response. Although TMB has good application prospects in the ICB treatment of other solid tumors, TMB still has some limitations. Many studies have turned to the development of other biomarkers closely related to TMB statuses, such as gene variation in the DNA damage response pathway and TP53/KRAS.

At present, integrating information about KRAS status and concurrent mutations into a comprehensive predictive model is a promising strategy to identify patients who might significantly benefit from, or possibly remain unresponsive to, immune checkpoint inhibitors (ICIs). However, the current body of data is yet insufficient to substantially influence treatment decisions. As it stands, definitive evidence delineating varied clinical outcomes with ICIs, contingent on the presence or absence of KRAS mutations, remains elusive.

Nonetheless, our research underscores the potential predictive value of KRAS mutations in determining responses to ICIs in cases of non-small cell lung cancer (NSCLC). Moving forward, a thorough investigation into the roles of KRAS mutations and their subtypes, alongside an analysis of drug response patterns and impacts on the immune system, will be pivotal in shaping the design of forthcoming trials, geared towards addressing the nuanced needs of this diverse patient population. In addition, the composition of the TME, including TILs, Tregs, and TAMs, are crucial for the immune response (24, 25). Furthermore, our data showed that B cell naive, T cell CD4 + memory activated, T cell regulatory, and other immune cells differ significantly among different KRAS mutated subtypes, which are associated with a suppressive immune environment for ICI therapy. Among them, Treg cells suppress antitumor immune responses, but the performance of Treg cells in the metabolically abnormal tumor microenvironment remains unknown (26). Regulatory T cells were capable of spontaneous and PD-L1 binding to block T cell-mediated anti-tumor immune responses before undergoing death (27).

Limitations of this study encompass: While our patient cohort consists of 103 individuals diagnosed with stage III-IV NSCLC and bearing KRAS mutations, additional cohorts from diverse centers would further validate our findings. To deepen our understanding of the intricate relationship between the KRAS mutant subtype and the tumor microenvironment (TME), more detailed mechanistic studies on how this mutation influences the TME are warranted.

In conclusion, we demonstrate that not all KRAS mutations are equivalent in predicting the efficacy of ICIs in patients with non-small cell lung cancer. At the same time, our data showed that different subtypes of KRAS mutations were significantly different in their association with TMB and TME compositions and the distribution of DNA damage repair defects. Our study suggests that selecting appropriate treatment modalities according to the subtype of patients with KRAS mutations may be a more desirable treatment selection strategy. In addition, the potential heterogeneity of KRAS mutations should be considered when evaluating KRAS as a biomarker for ICIs.

## Data availability statement

The datasets presented in this study can be found in online repositories. The names of the repository/repositories and accession number(s) can be found in the article/**Supplementary Material**.

## Author contributions

QW: Conceptualization, Data curation, Formal Analysis, Investigation, Methodology, Project administration, Validation, Writing – original draft, Writing – review & editing. ZT: Conceptualization, Data curation, Formal Analysis, Investigation, Methodology, Writing – original draft. CL: Supervision, Validation, Writing – review & editing. XL: Conceptualization, Formal Analysis, Funding acquisition, Investigation, Methodology, Project administration, Supervision, Writing – review & editing. CS: Conceptualization, Data curation, Formal Analysis, Funding acquisition, Investigation, Methodology, Project administration, Resources, Supervision, Validation, Writing – review & editing.

## Supplementary material

Supplementary Figure 1
**(A–E)** Violin plots illustrating variations in the expression levels of different immune checkpoint markers across distinct KRAS subtypes.

Supplementary Figure 2
**(A–G)** Gene Set Enrichment Analysis (GSEA) showcasing the distribution of differentially expressed genes across four distinct KRAS mutation subtypes.

Supplementary Figure 3 Graphical representation of IC50 drug response, showing the disparate responses of different KRAS mutation subtypes to various pharmaceutical agents.
