# Supplementary figures and images for: Evaluating distinct KRAS subtypes as potential biomarkers for immune checkpoint inhibitor efficacy in lung adenocarcinoma
Source: Front Immunol. 2023 Oct 24;14:1297588. doi: 10.3389/fimmu.2023.1297588 (PMC10635421; doi:10.3389/fimmu.2023.1297588)

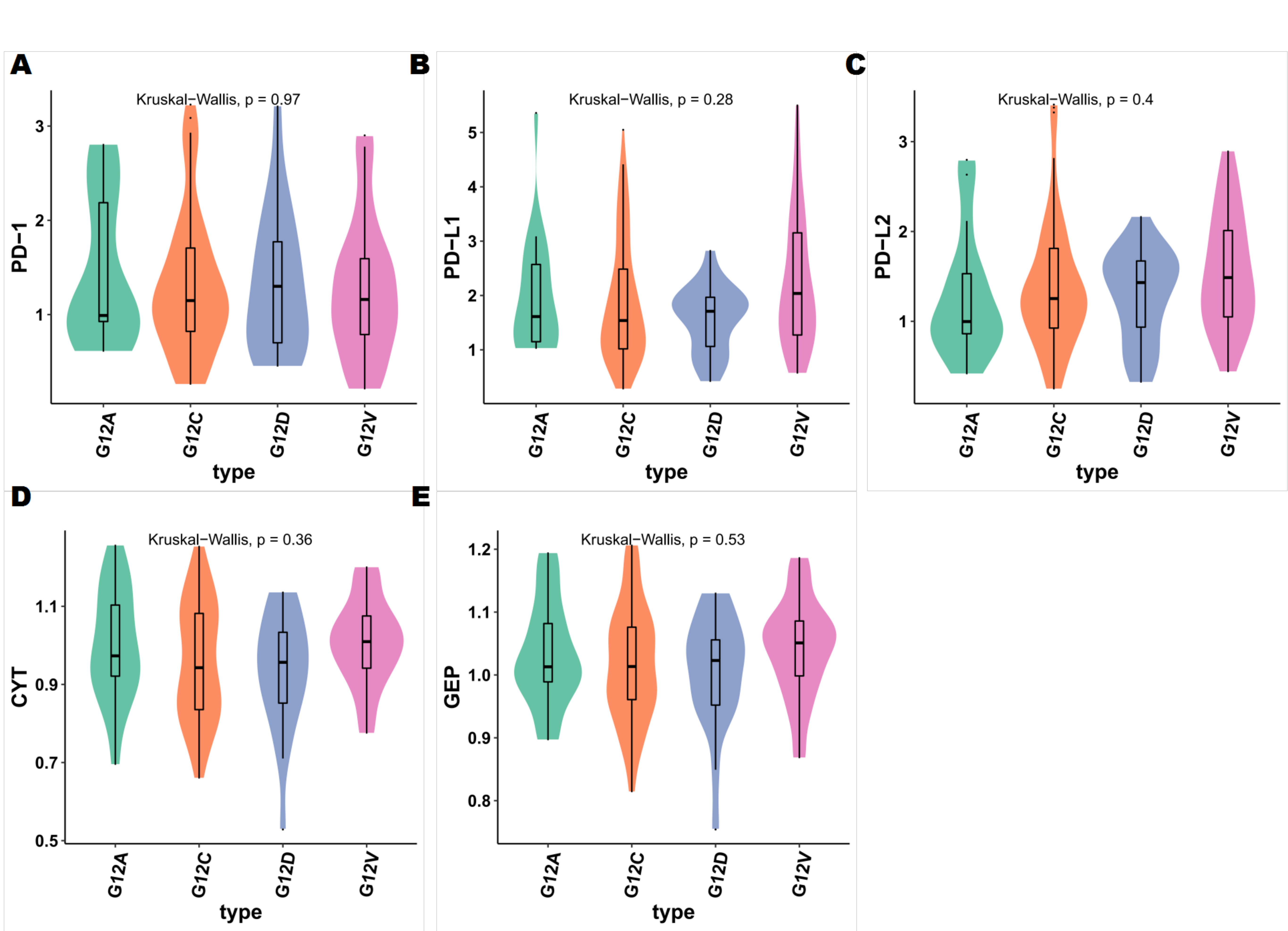

Supplement: Supplementary Figure 1 — (A–E) Violin plots illustrating variations in the expression levels of different immune checkpoint markers across distinct KRAS subtypes. [file Image_1.tif]

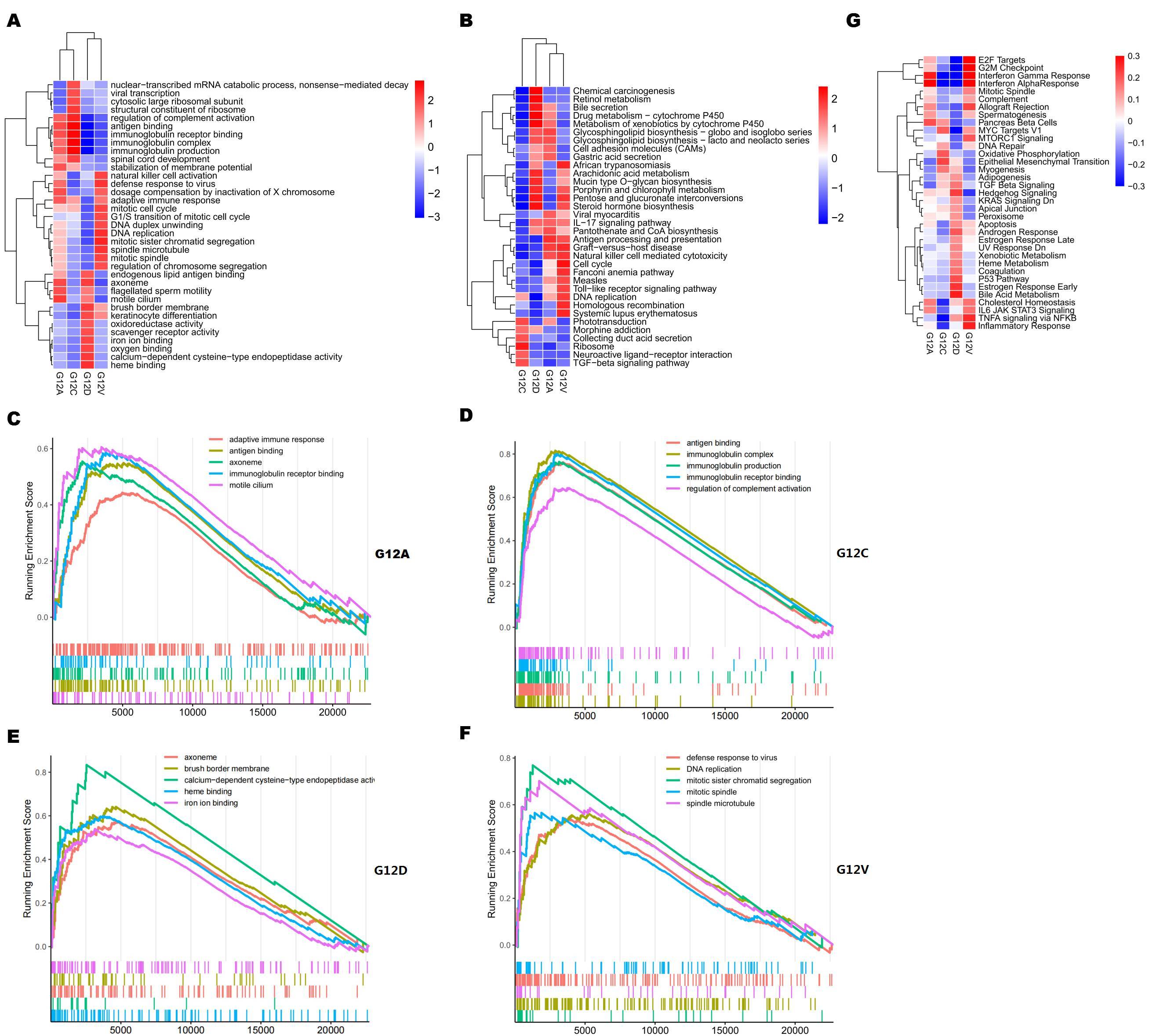

Supplement: Supplementary Figure 2 — (A–G) Gene Set Enrichment Analysis (GSEA) showcasing the distribution of differentially expressed genes across four distinct KRAS mutation subtypes. [file Image_2.jpeg]

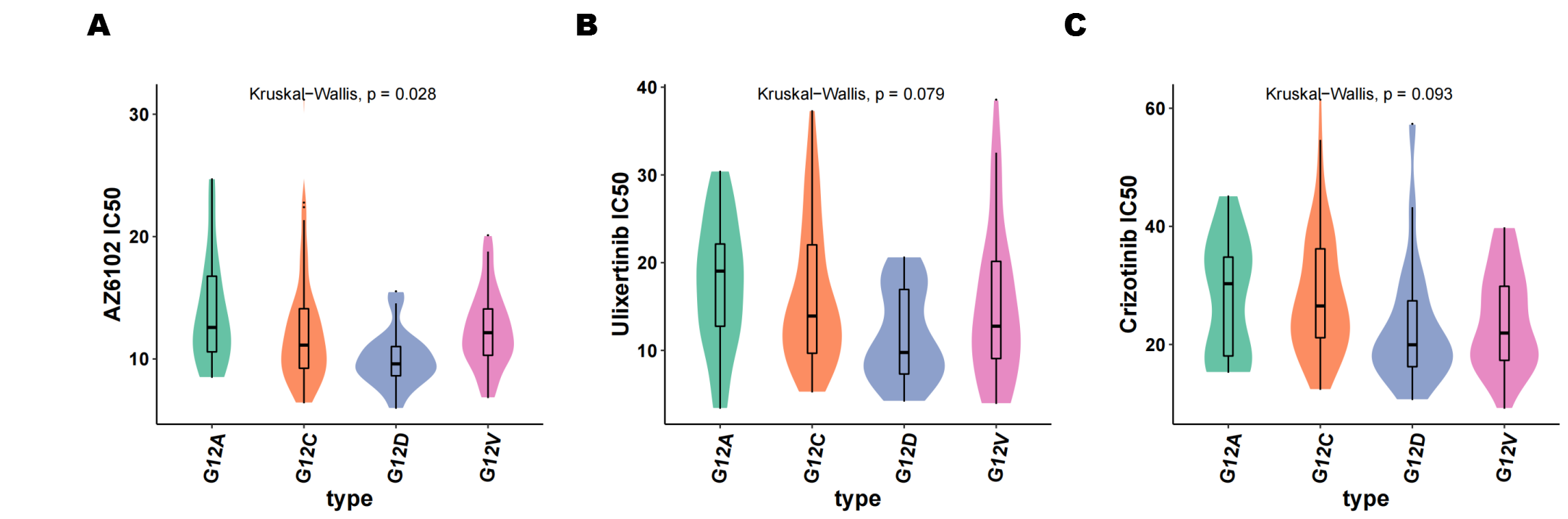

Supplement: Supplementary Figure 3 — Graphical representation of IC50 drug response, showing the disparate responses of different KRAS mutation subtypes to various pharmaceutical agents. [file Image_3.tif]
